# Association Between Body Mass index and Prevalence of Multimorbidity in Low-and Middle-income Countries: A Cross-Sectional Study

**DOI:** 10.5530/ijmedph.2016.2.5

**Published:** 2016-04

**Authors:** Sutapa Agrawal, Praween Kumar Agrawal

**Affiliations:** 1 Public Health Foundation of India, New Delhi NCR, INDIA; 2 United Nations Children’s Fund, New Delhi, INDIA

**Keywords:** Overweight/obesity, Non communicable disease multimorbidity, Chronic Conditions, Low and middle income countries, WHO-SAGE

## Abstract

**Background:**

Chronic diseases are increasingly becoming a health burden in terms of both morbidity and mortality in low and middle-income countries (LMICs). The role of body mass index (BMI) especially overweight and obesity in the prevalence of multimorbidity, the occurrence of two or more chronic conditions, is understudied in LMICs where two thirds of the world’s obese individuals reside. We estimated the association between BMI and prevalence of chronic non communicable disease multimorbidity in six LMICs.

**Methods:**

Cross-sectional data of total of 40,166 participants from China (n=13,970), India (10,915), Mexico (2,4 26), Russia (3,892), South Africa (4,000) and Ghana (4,971), aged 18 years and above included in the WHO Study on Global Ageing and adult health (SAGE), 2007–2010 were analyzed. Multimorbidity was measured as the simultaneous presence of two or more of the nine chronic conditions including angina pectoris, arthritis, asthma, chronic lung disease, diabetes mellitus, hypertension, stroke, depression, and vision impairment. Multivariable logistic regression models were fitted to test for associations between overweight/obesity and prevalence of non communicable multimorbidity after adjusting for age, sex, rural/urban residence, education, marital status, occupation, household wealth, tobacco smoking, alcohol drinking, fruits and vegetable intake and health insurance status. Data were analyzed country wise as well as pooled together to give overall LMIC estimates.

**Results:**

The mean BMI was 24.4 [±7.3SD] in the pooled countries, being as low as 20.8 [±8.0 SD] in India to 23.4 [±6.3 SD] in Ghana, 23.9 [±4.9 SD] in China, 28.4 [±5.4 SD] in Mexico, 28.6 [±6.3 SD] in Russia, to as high as 30.5 [±12.0 SD] in South Africa. The prevalence of overweight was 13% and obesity was 24% in the pooled sample. The prevalence of non communicable disease multimorbidity was 23% in the pooled sample of six countries–the highest being in Russia (50%), followed by Mexico (27%), India (24%), Ghana (23%), South Africa (32%) and China (22%). The prevalence of multimorbidity was 37% among obese population and 27% among overweight population in the pooled sample–highest prevalence was in Russia (59% among obese; 45% among overweight) and lowest in Ghana (28% among obese; 23% among overweight). Being obese (OR:5.78;95%CI:3.55–9.40;p<0.0001) was associated with significantly higher likelihood of having multimorbidity as compared to normal weight category in the pooled sample. The likelihood of multimorbidity among obese were almost ten times higher in Russia (OR:9.90;95%CI:3.90–25.17;p=<0.0001), seven times higher in China (OR:7.06;95%CI:2.47–20.21;p=0.003), six times higher in Ghana (OR:5.61;95%CI:1.21–26.02;p= 0.007) and five times higher in South Africa (OR:4.66;95%CI:2.16–10.08;p=0.005). Non-significant but positive association were also observed in case of India and Mexico. The likelihood of multimorbidity was more than two times higher among overweight population in India (OR:2.33;95%CI:1.35–4.02;p=0.003) and pooled countries (OR:1.47;95%CI:1.05–2.07;p=0.004) while non-significant but positive association were also observed in case of China, Russia, and Ghana.

**Conclusions:**

The prevalence of non communicable disease multimorbidity in the LMICs is high, one and half times higher in obese than in normal weight individual. Obesity was independently associated with the occurrence of multimorbidity in the six LMICs. These findings may be vital for public health surveillance, prevention and management strategies for non communicable disease multimorbidity in the LMICs.

## Introduction

Chronic or non-communicable diseases (NCD) are increasingly becoming a major health burden both in terms of morbidity and mortality especially in low and middle-income countries (LMICs). Multimorbidity, which is the state of simultaneous occurrence of two or more chronic conditions within an individual without any single predominant condition, is an emerging issue in public health agenda in LMICs due to its increasing prevalence, impact on individual health status, and the financial impact on the health care system.^1^–^15^ People with multimorbidity are considered to be at increased risk of receiving low individual and medical care,^7^–^9^ more frequent and longer hospitalizations,^8^ higher health care costs including out of pocket expenditure,^7^,^9^,^10^,^16^ and increased use of polypharmacy,^6^ with the potential for adverse drug effects.^8^,^9^ Global estimates of the prevalence of multimorbidity vary from 17% to over 90%,^2^,^11^,^17^,^18^ with the highest prevalence in developed countries where about 1 in 4 adults experience multimorbidity, with half of older adults having 3 or more chronic conditions.^2^,^11^ It is expected that multimorbidity will soon become a norm rather than the exception in both developed as well as developing nations.^12^ However, most public health systems as well as medical treatment strategies, even in developed nations are still designed and implemented as if diseases occur in isolation.^13^

Excess weight or overweight and obesity are considered an important predecessor to NCD multimorbidity,^19^ and an important risk factor for future morbidity.^20^ Epidemiological studies conducted in developed nations have found that multimorbidity was more commonly prevailing in ageing and obese patients,^9^,^21^,^22^ and even in the general population.^20^ A cohort study conducted in UK found that 32% of multimorbidity was attributable to overweight and obesity among patients registered in a primary care.^23^ Excess bodyweight was also associated with increased frequency of many other long-term conditions, including type 2 diabetes, cardiovascular diseases and musculoskeletal problems.^24^–^27^ The association of overweight and obesity with multimorbidity thus qualifies for evaluation in LMICs where two thirds of the world’s obese individuals reside and an epidemiological transition is already underway. An understanding of this association may offer insight into the contribution of overweight/obesity to the burden of multimorbidity in the general population and the potential for prevention of multimorbidity in LMICs. The aim of this study is to evaluate the prevalence of NCD multimorbidity by overweight/obesity status based on a list of nine chronic conditions and investigate the association between body mass index and prevalence of multimorbidity in the six LMICs. We hypothesized that prevalence and risk factors for multimorbidity is more common in the overweight/obese than non-overweight/obese population in the LMICs.

## Methods

### Participants and Data

We used data from the WHO Study on Global AGEing and Adult Health (SAGE) wave 1 (2007–2010). SAGE is a longitudinal ageing and health study with nationally representative samples of adults (>18 years) in six LMICs — China, India, Ghana, Mexico, Russian Federation and South Africa.^28^ The countries were chosen to represent different geographic regions of the world, levels of economic development and stages in the demographic and health transition and include the two most populous countries of the world.^29^ SAGE is also designed to provide results that are comparable to ageing studies in high-income countries, such as the US Health and Retirement Study (http://hrsonline.isr.umich.edu/), the English Longitudinal Study on Ageing (www.ifs.org.uk/elsa/) and the Collaborative Research on Ageing in Europe (COURAGE in Europe) Project (www.courageproject.eu/) in three countries.^28^

The SAGE study sampling plans used multistage clustered design samples drawn from an updated frame,^28^ in all countries, except for Mexico. Households were classified into one of two mutually exclusive categories: (1) all persons aged 50 years and older were selected from households classified as ‘50+households’; and (2) one person aged 18–49 years was selected from a household classified as an ‘18–49 household’. The arrangement in Mexico was similar, but included supplementary and replacement samples to account for losses to follow up in selected sampling units since Wave 0.^28^ The sample in India is also representative at the sub-national and sub-state levels for the selected states. Each household and individual is assigned a known non-zero probability of being selected.^28^ Household and individual weights were post-stratified to weight up to population distributions by age and sex in each country. Detailed description of study and sample design is documented elsewhere.^28^,^30^

Face-to-face interviews were used to collect household and individual level data, by using standardised survey instruments in native language of respondents and protocols, covering a broad range of topics, including health and its determinants, disability, subjective, emotional and financial wellbeing, health care utilisation and health systems responsiveness.^28^ One household questionnaire was completed for each selected household in face-to-face interviews and individual questionnaires were collected from one randomly selected individual aged 18–49 years and all individuals aged over 50 years, including by proxy where an individual was unable to complete the questionnaire.^28^ Individual response rates are as follows: 53% Mexico, 68% India, 75% South Africa, 81% in Ghana, 83% Russia and 93% in China.^28^ A physical examination was used to collect height, weight, waist circumference and blood pressure. The SAGE dataset is described in full elsewhere and the questionnaires are available in public domain at http://www.who.int/healthinfo/sage/cohorts/en/index2.html.

### Outcome measures

#### Chronic conditions and multimorbidity

We defined multimorbidity as concurrent occurrence of two or more chronic health conditions in the same individual. For the purpose of the study the following nine chronic conditions were analysed:

Angina pectoris, arthritis, asthma, low visual acuity, chronic lung disease (includes emphysema, bronchitis, chronic obstructive pulmonary disease), diabetes (excluding diabetes associated with a pregnancy), stroke, hypertension and depression. Of these nine conditions, diabetes mellitus and stroke were assessed through a question about ever being diagnosed with the disease by a health professional. The specific question was, *“Have you ever been told by a health professional/doctor that you have (disease name)?”* The prevalence of angina pectoris, arthritis, asthma, and chronic lung disease was derived from a set of symptom based questions, combined with a validated diagnostic algorithm as described in Arokiasamy *et al*.^31^ Additionally, we have used of treatment/medication received in the 12 months prior to interview which was indicative of a diagnosis and was included in prevalence estimates for the above described chronic conditions. We used a symptom based reporting and diagnostic algorithm for the prevalence of angina pectoris, arthritis, and asthma. The assessment of hypertension and visual acuity was based on direct physical examination undertaken at the time of interview.^31^ The prevalence of hypertension was based blood pressure (systolic and diastolic) measured three times on the right arm/wrist of the seated respondent using an automated recording device (OMRON R6 Wrist Blood Pressure Monitor, HEM-6000-E, Omron Healthcare Europe, B.V., Hoofddorp, The Netherlands).^32^ An average of the second and third of three total readings was used as the outcome in this study. In accordance with WHO/ISH guidelines for the management of hypertension,^33^ the limit for high systolic blood pressure was 140 mm/hg or above, and for diastolic blood pressure 90 mm/hg or above.^34^ An individual was considered to be hypertensive if average systolic or diastolic blood pressure readings exceeded either of these thresholds or they reported current treatment for hypertension.^31^ Visual impairment is associated with functional limitations and lowered well-being, and also affects health related quality of life through its effect on self-care and treatment-seeking behaviour.^35^ We measured visual acuity in this study for both near and distance vision in each eye using a tumbling “E” logMAR chart.^36^ Measured near and distance visual acuity was classified into normal vision (0.32–1.6 decimal) and low vision (0.01–0.25 decimal).^37^ We categorised respondent had low vision if they had either low near or distance vision in both eyes.^31^

We used a self-reported symptom-based depression in the past 12 months for inclusion in the multimorbidity criteria. The symptomatic depression items were assessed based on the World Mental Health Survey version of the Composite International Diagnostic Interview.^38^ The diagnosis of depression was based on the International Classification of Diseases, 10th revision (ICD-10), diagnostic criteria for research (DCR) for depressive episodes,^39^ which were derived from an algorithm that took into account respondents reporting symptoms of depression during the past 12 months.^40^ Participants endorsed at least 4 of 10 depressive symptoms lasting 2 weeks most of the day or all of the day. According to the ICD–10–DCR criterion B, at least two of the following three symptoms needed to be present in order to be diagnosed as depressive: depressed mood, loss of interest, and fatigability.^40^ Additionally, the respondents who responded positively to the question, *‘Have you been taking any medications or other treatment such as attending therapy or counselling sessions for depression during the last 12 months?’* was also added to the symptom-based depression.^31^

#### Body Mass Index

Apart from the self-reported height and weight data, trained SAGE surveyors had obtained participant height (cm) and weight (kg) measurements using standard procedures. Specifically, respondents were asked to wear a single layer of clothing and remove their shoes; participant height was then measured to the nearest 0.1 cm using a stadiometer and weight was recorded to the nearest 0.1 kg using a weighing scale. These were then used to calculate individual body mass index (BMI; kg/m^2^) where both values were non-missing. BMI values were classified into categories for each individual based on established WHO cut-offs for BMI, which included four categories: underweight (<18.5 kg/m^2^), normal (18.5–24.9 kg/m^2^), overweight (25.0–29.9 kg/m^2^), and obese (≥30 kg/m^2^).^41^ For data presented by obesity status, obesity itself was not included as a chronic condition in the definition of multimorbidity.^20^

#### Indicators of socioeconomic status and control variables/confounders

Socio-economic and demographic factors included in our analysis were age groups (18–29, 30–39, 40–49, 50–59, 60–69, 70+ years); sex; marital status (married, not married, others (includes separated, divorced and widowed)), education (no education, primary school or less, secondary school, tertiary or higher education); place of residence (rural, urban); quintiles of household wealth/assets, (Q1 lowest to Q5 highest), occupation (public sector, private sector, self-employment, informal sector), tobacco smoking (never, occasionally, daily), ever alcohol intake (no, yes), fruits and vegetables intake (no or insufficient (<5 servings/day), sufficient (>5 servings/day) and health insurance status (without health insurance, with health insurance).

Household wealth status was already provided in dataset,^42^ derived using WHO standard approach to estimating permanent income from survey data on household ownership of durable goods, neighbourhood and dwelling characteristics, and access to water, sanitation, electricity etc.^43^ Fruit and vegetable consumption was assessed with the questions ‘How many servings of fruit do you eat on a typical day?’ and ‘How many servings of vegetables do you eat on a typical day?’ Researchers were trained to show all respondents a nutrition risk factor card that indicates both in writing and in pictures general categories, amounts, and examples of fruits and vegetables in an attempt to standardise the serving size and number of servings reported.^44^ Insufficient fruits and vegetable consumption were defined as less than five servings of fruits and/or vegetables a day.^44^ Tobacco and alcohol consumption was included as a part of lifestyle behaviour. Lifetime tobacco used was assessed with the question ‘Have you ever smoked tobacco or used smokeless tobacco?’ Lifetime tobacco users were asked ‘Do you currently use (smoke, sniff, or chew) any tobacco products such as cigarettes, cigars, pipes, chewing tobacco, or snuff?’ The response options were ‘Yes, daily’, ‘Yes, but not daily’, and ‘No, Not at all’.^45^ These questions are based on the WHO Guidelines for Controlling and Monitoring the Tobacco Epidemic.^46^ Lifetime alcohol use was assessed with the question ‘Have you ever consumed a drink that contains alcohol (such as beer, wine, spirits, etc.)?’ Response options were ‘Yes’ or ‘No, never’.^45^ Lifetime alcohol users were asked about current (past month) alcohol use, and current alcohol users were asked ‘During the past 7 days, how many drinks of any alcoholic beverage did you have each day?^47^

#### Statistical analyses

We calculated the pooled estimate using data from all countries, but also ran separate analyses for each country. The prevalence of multimorbidity was estimated in relation to BMI status, age, sex, place of residence, marital status, education level, occupation, household wealth, tobacco smoking, alcohol intake, fruits or vegetable intake and health insurance status, and other factors as discussed above. Categorical variables are presented as absolute and relative frequencies. Associations between categorical variables were tested by the calculation of the chi-square test. Multivariable logistic regression models were then fitted to test for associations between overweight/obesity and NCD multimorbidity after adjusting for the confounders discussed above. Odds ratios (OR) and 95% confidence intervals (CI) were calculated for each risk-factor using logistic regression adjusted for survey design. For a simple explanation of odds ratios, see Grimes and Schultz.^48^ BMI category was the principal predictor while the outcome was presence/absence of multimorbidity (a dichotomous variable based on the number of conditions). For the purpose of the study, we excluded those with missing values for outcome and independent variables (9.2% of the sample). We tested for possible multicollinearity for covariates adjusted for in our analysis through correlation matrix and found the multicollinearity were all less than 0.5, indicating that the assumption of reasonable independence among predictor variables and the outcome variable was met.^49^ The absence of multicollinearity and plausible interactions among variables were tested to ensure the robustness of the regression model.^49^ In all data analysis, sampling weights were used to account for the complex, multistage design of the SAGE survey.^28^ We performed the statistical analyses using STATA 10.1 (StataCorp, College Station, Texas). It is important to mention here that, the pooled data have a hierarchical structure with individuals nested within regions which are in turn nested within countries.^50^ The pooled analysis combines time series for several cross-sections.^31^ However, particular care should be taken when interpreting the pooled data since estimates from a model that includes a full set of interactions between individual characteristics and the country dummies are not equivalent to the estimates derived from distinct country regressions,^16^,^31^,^50^ because the residual error variance is constrained to be the same across countries in the former case but not in the latter.^51^–^52^

The WHO-SAGE study wave 1 study has a total of 36,354 individual respondents aged 50 years and above and 7,961 individuals aged 18–49 years in a total of six nations. The individual level response rates for each country were as follows: China (93%), Ghana (81%), India (68%), Mexico (53%), Russia (83%) and South Africa (75%). This high response rate in the SAGE countries were achieved mostly for two reasons: the majority of the respondents were participants in the World Health Survey 2002–2004 and followed up and the local institutions applied concerted efforts in collaboration with local partners to improve survey response which included conducting a minimum of three revisits to households. We did a secondary analysis of nationally representative cross-sectional data of 44,715 participants aged 18 years and above included in the WHO Study on Global AGEing and adult health (WHO SAGE), 2007–2010. The final sample size analysed for the six countries are as follows: China (n = 15,048), India (n=12,198), Mexico (n = 2,725), Russian Federation (n = 4,946), South Africa (n = 4,227) and Ghana (n = 5,571).

#### Ethics approval

The WHO-SAGE study received human subjects testing and ethics council approval from research review boards local to each participating country (China: China Centre for disease control and prevention ethical review committee; Ghana: University of Ghana Medical School Ethics and Protocol Review Committee; India: Institutional Review Board, International Institute for Population Sciences, India; Mexico: Comisión de Ética en Investigación, Instituto Nacional de Salud Publica; Russia: Department of Prophylactic Medicine, Russian Academy of Medical Sciences; South Africa: Human Sciences Research Council Ethics Committee, South Africa), and overall from the WHO Ethical Review Committee. Written Informed consent was obtained from each respondent prior to interview and examination. Our study is a secondary analysis of SAGE de-identified data which is available in public domain and does not require ethics committee approval.

## Results

### Participant characteristics

Table 1 shows the characteristics of the study sample of each country. The distribution of BMI status in the pooled sample was as follows: 13% obese, 24% overweight, 49% normal and 13% underweight. The distribution of obese individual was highest in South Africa (43%), followed by 33% each in Mexico and Russia, while it was lowest in India (3%), China (6%) and Ghana (10%). Two-fifth of the sample population in Mexico and Russia were overweight, followed by 28% in South Africa and 27% in China, 19% in Ghana and 11% in India. The mean BMI was 24.4[±7.3] in the pooled countries, being as low as 20.8[±8.0SD] in India to 23.4[±6.3 SD] in Ghana, 23.9[±4.9 SD] in China, 28.4[±5.4 SD] in Mexico, 28.6[±6.3 SD] in Russia, to as high as 30.5[±12.0 SD] in South Africa. Women respondents constitutes 56% of the total sample in the overall six LMICs, the proportion ranging from almost half in Ghana and China to more than 60% in India, Mexico and Russia. The respondents were drawn largely from rural areas in India (74%), Ghana (59%) and China (51%) while rural respondents consisted of a smaller proportion of the sample in Mexico (26%), Russia (25%) and South Africa (33%). A majority of the sample population were married at the time of survey in China (83%), India (78%), 60% in Ghana, 59% in Mexico and 57% in Russia. Half of the sample respondents were not educated in Ghana, more than two-fifths in India, one fourth each in China and South Africa while it is only 1% in case of Russia. A majority were a public sector employee in Russia (86%) whereas 80% were self employed in Ghana. Highest daily current tobacco users are in India (86%), followed by 75% in China, 64% in South Africa, 56% in Russia, 38% in Ghana and 32% in Mexico. Ever alcohol use was highest in Russia (75%), while it was lowest in India (13%). Except china (89%), the percentage distribution of respondents reporting sufficient intake (>5servings/day) of fruits and vegetable was low (ranges from 13% in India to 33% in Ghana). The distribution of nine self reported chronic conditions were similar in the six countries with a higher number of participants reporting high BP, diabetes and arthritis than other conditions except in Mexico, where other conditions were more common than diabetes. In four of the countries low visual acuity was more commonly reported than diabetes. In the pooled frequencies, the top three most common prevalent chronic conditions were hypertension (34%), arthritis (19%) and low visual acuity (14%) followed by chronic lung disease (10%), angina (8%) and depression (7%).

### Prevalence of multimorbidity by BMI categories and other characteristics

Table 2 shows the mean number of chronic conditions and prevalence of multimorbidity by body mass index and other characteristics. The mean number of chronic conditions ranged from 3.0 in Russia to 2.4 in Ghana and it was 2.6 in the pooled sample. The prevalence of multimorbidity was 23% in the pooled sample of six countries–the highest being in Mexico (56%), followed by Russia (46%) South Africa (32%), India (24%), Ghana (23%) and China (21%). The prevalence of non communicable disease multimorbidity was 23% in the pooled sample of six countries–the highest being in Russia (50%), followed by Mexico (27%), India (24%), Ghana (23%), South Africa (32%) and China (22%). The prevalence of multimorbidity was 37% among obese population and 27% among overweight population in the pooled sample–highest prevalence was in Russia (59% among obese; 45% among overweight) and lowest in Ghana (28% among obese; 23% among overweight).

### Association between BMI status and multimorbidity

Table 3 gives the adjusted odds ratios showing the association between BMI status and non communicable disease multimorbidity in the six countries as well as in the pooled sample. Obese were significantly higher likelihood of having multimorbidity as compared to normal weight category in the pooled sample (OR:5.78; 95%CI:3.55–9.40; p<0.0001). The likelihood of multimorbidity among obese were almost ten times higher in Russia (OR:9.90;95%CI:3.90–25.17;p=<0.0001), seven times higher in China (OR:7.06;95%CI:2.47–20.21;p=0.003), six times higher in Ghana (OR:5.61;95%CI:1.21–26.02;p=0.007) and five times higher in South Africa (OR:4.66;95%CI:2.16–10.08;p=0.005). Non-significant but positive association were also observed in case of India and Mexico. The likelihood of multimorbidity was more than two times higher among overweight population in India (OR:2.33;95%CI:1.35–4.02;p=0.003) and pooled countries (OR:1.47;95%CI:1.05–2.07;p=0.004) while non-significant but positive association were also observed in case of China, Russia, and Ghana.

### Discussion

#### Main findings

In this study we evaluated the association of overweight and obesity with the prevalence of non communicable disease multimorbidity (nine chronic conditions encompassing angina pectoris, arthritis, asthma, chronic lung disease, diabetes mellitus, hypertension, stroke, low visual acuity and depression) by using nationally representative data from the WHO SAGE in six low and middle-income countries. Multimorbidity was found to be strongly associated with obesity which is consistent with our hypothesis as well as findings from previous studies from developed countries data.^19^,^23^,^53^ The significant positive association between overweight/obesity and NCD multimorbidity in the LMICs was robust after controlling for age, sex, rural/urban residence, education, occupation, marital status, household wealth, tobacco smoking, current alcohol intake, fruits and vegetable intake and health insurance status. The prevalence of multimorbidity in the adult population was quite high (28%) in the pooled sample of six countries and varied markedly, from 21% in China to 56% in Mexico. The mean number of chronic conditions was 1.7 in the pooled sample of six countries and ranged from 1.4 in China to 2.3 in Mexico. Prevalence of multimorbidity was twice among obese than in overall population (44% vs 23%) in the pooled sample of six countries. The prevalence of multimorbidity in obese population was two times higher than among normal weight population and nearly one and half times higher among overweight population. Women had consistently higher rates of multimorbidity than men in all the six SAGE countries we studied.

It is important to note that the inclusion of obesity in multimorbidity indices has been debated in the western literature.^54^–^55^ Nevertheless, given that fact the prevalence of obesity is increasing and that it is associated with increased risk of adverse health states, including all-cause mortality,^56^ there is a need for its inclusion in multimorbidity indices. In a world where obesity is rapidly on the rise, it’s inclusion also makes more sense in terms of health system planning and when view in the prism of different metabolic of many pharmacological products in the obese. Our study showed that obesity prevalence positively associated with multimorbidity, with obesity being more common in individual with higher number of chronic conditions in all the six countries we considered.

#### Policy Implications of findings

This is a study of data from six populous LMICs currently undergoing nutrition and epidemiological transition characterised by rapid increases in obesity and non-communicable disease.^26^ Our analyses, provide national estimates of the prevalence of multimorbidity amongst adults according to their BMI status and the association of BMI with prevalence of non communicable disease multimorbidity. Indeed China, Russia, India and Mexico are four of the countries with the highest number of obese individuals in the world, with China and India estimated to contain 15% of the world’s obese population.^57^ Our study is the first to quantify the relationship between excess weight and non-communicable disease multimorbidity in six LMICs. Our study shows that, health care providers should be aware of the likelihood of multiple diseases in overweight and obese patients and measurement of BMI and formal recognition of obesity should be used to identify patients at high risk of multimorbidity by policy makers and programme managers. Weight loss can lead to reductions in the incidence of diabetes and remission of symptoms in obese patients.^58^ Given that both obesity and diabetes are risk factors for the onset of cardiovascular disease,^23^ it is possible that weight reduction could also impact on the incidence of other conditions such as stroke or myocardial infarction in these patients.^59^ The inclusion of BMI to identify and monitor obesity should thus be a priority for health care, along with weight management intention and behaviour and targeted control of other risk factors such as hypertension and diabetes.^23^ Such changes in medical practice in LMICs could potentially reduce the onset and burden of multimorbidity and should be effectively prioritise in the background of a growing epidemic of obesity.

#### Strengths and Limitations

This study uses large, nationally representative data from six populous low- and middle-income countries experiencing rapid economic growth, urbanisation and increasing NCD risk,^49^–^50^ and the distribution provides robust cross-national level estimates of our key variables (50). The SAGE survey data were collected using consistent tools and measures (31), including objective measures of anthropometry and blood pressure, allowing robust cross country comparisons.^28^ Taking into account the possible bias introduced by disease prevalence derived from self-reported physician diagnosis,^60^–^63^ WHO-SAGE incorporated a number of alternate methods of estimating disease – using a mixture of self-reported diagnosis cum validated symptom reporting-based diagnostic algorithms, and objective health measurements criterias.^28^–^31^ This makes the findings that obesity is strongly associated with multimorbidity all the more remarkable and of major importance in public health and policy terms in LMICs.

The findings from this study should also be viewed in light of important limitations. First, the use of survey data and self-reported measures of NCDs (for e.g., diabetes and stroke) are a potential source of bias including social desirability bias, the potential for greater under-reporting of NCDs in persons from lower socioeconomic backgrounds,^64^–^66^ as well as error or over-reporting of conditions. Self-reported chronic disease status is subject to self-declaration bias also due to under-reporting of diagnosis or forgetfulness.^67^–^69^ Studies found that lower socioeconomic groups had less screening and knowledge of cardiovascular risk factors, whereas those with the knowledge were more likely to make healthy behavioural changes,^70^ and educational attainment and health literacy can modify the NCDs and risk factors in LMICs.^71^–^72^ A recent study using the same data base indicated that socioeconomic gradients in NCD prevalence qualitatively differed within and between countries by type of prevalence measurement, specific NCDs and socioeconomic indicators, and thus NCDs as a category cannot be considered as diseases of affluence or of poverty.^73^ Some individuals who report having multimorbidity may essentially be reporting a single chronic condition and its symptom, e.g. arthritis and chronic pain.^31^ This may lead to overestimation of the true prevalence of multimorbidity.^10^ We however, used validated diagnostic algorithms to neutralise this bias for all chronic conditions wherever possible in the data set. Another limitation of this study is the absence of an indicator of disease severity as we assessed multimorbidity by counting the number of NCDs without applying any weights to account for severity of conditions.^2^,^31^ We have used a count of chronic conditions as a measure of multimorbidity, which implies that each of the diseases has equivalent impact on an individual.^16^,^31^ The allocation of an equal weight to all chronic conditions fundamentally assumes that the listed chronic conditions are the same though in reality, the effects of multimorbidity on various domains of health are likely to depend on disease severity, the unique combination of diseases, and access to treatment and support.^31^ A further step may be to incorporate a severity weight to the chronic conditions as suggested in by various studies using this data base.^10^,^16^,^31^,^50^ Although SAGE asked about nine common NCDs, the list is not exhaustive and some common NCDs were not included.^72^ For e.g., among all other heart conditions/diseases only angina alone was included in the survey but others were not included. We may be missing some higher burden conditions, such as dementia and cancers, which could have resulted in an underestimation of the prevalence and impacts of multimorbidity.^73^ However, a number of studies have analyzed multimorbidity using a smaller number of diseases, usually less than 10, due to data limitations in LMICs.^3^,^10^,^50^ Nevertheless, the prevalence found in our study is remarkable and actually might be more alluring if all health conditions were captured in a developing country setting. Additionally, the WHO-SAGE questionnaire did not include detailed information on diet composition; these analyses therefore did not control for individual dietary factors. However, we could use the portions of fruit and vegetable consumption which was provided in the dataset, which is the proxy for dietary intake. Ethnicity and religious affiliation were also not included in the study due to the large number of missing values generated for each country. Another major limitation of the data is that we were not been able to take into account the potential side effect of weight gain due to medication use, which might be crucial. Also, many chronic conditions will reduce mobility and ability to exercise, which in turn may lead to increased weight which we could not confirm from the data. Finally, the cross-sectional study design limits causal interpretation of our findings and uncontrolled confounding such as genetic and environmental factors, family history of diseases, physical activity and other lifestyle factors cannot be excluded as an explanation for the association we studied. There is an urgent need to examine the association between overweight/obesity and multimorbidity in depth using prospective cohort study designs in a developing country setting.

## Conclusions

With the study samples drawn from six LMICs with different levels of development and healthcare system largely built on a social medical insurance system, we evaluated the prevalence of multimorbidity and provided an in-depth analysis of obesity and its association with non communicable disease multimorbidity and other factors, which are, in general, similar to other developed countries. Obesity was an important independent predictor of the occurrence of multimorbidity in this population. The findings are important for public health and policy makers who wish to monitor the obesity epidemic in LMICs. This is particularly relevant in light of the WHO 25x25 monitoring framework in which the prevalence of obesity is one indicator. Our study provides further evidence for policies and targeted interventions to tackle the growing burden of obesity along with non communicable disease multimorbidity. These findings highlight the importance of recognising the issue of obesity to tackle non communicable disease multimorbidity. Further, strategies to manage obesity may be relevant for prevention and management of multimorbidity in LMICs.

## Figures and Tables

**Table 1:** Socioeconomic and demographic characteristics of the respondents (18 years and above) included in WHO-SAGE 2007–2010 by country.

Characteristics	China [n=15048]	India [n=12198]	Mexico [n=2725]	Russia [n=4946]	South Africa [n=4227]	Ghana [n=5571]	Pooled countries [n=44715]
**BMI status**							
Normal (18–24.9kg/m^2^)	62.6[8744]	51.4[5610]	25.8[626]	25.2[979]	26.0[1039]	56.9[2827]	49.4[19825]
Underweight (<18.5kg/m^2^)	4.6[638]	34.8[3795]	0.9[21]	1.0[40]	3.9[155]	14.1[698]	13.3[5347]
Overweight (25.0–29.9kg/m^2^)	27.4[3823]	10.8[1174]	40.7[986]	41.0[1594]	27.7[1106]	19.0[945]	24.0[9628]
Obese (≥30kg/m^2^)	5.5[767]	3.1[336]	32.5[787]	32.9[1279]	42.5[1700]	10.0[499]	13.4[5368]
Mean BMI [±SD]	23.9[±4.9]	20.8[±8.0]	28.4[±5.4]	28.6[±6.3]	30.5[±12.0]	23.4[±6.]	24.4[±7.3]
Number of cases	13972	10915	2420	3892	4000	4969	40168
**Age**							
18–29	1.4[217]	15.0[1823]	2.1[57]	2.0[98]	2.4[103]	2.5[139]	5.5[2437]
30–39	3.4[511]	14.3[1745]	6.4[175]	3.0[147]	3.0[127]	5.7[316]	6.8[3021]
40–49	6.1[913]	12.1[1480]	7.1[192]	3.5[172]	3.7[155]	6.9[384]	7.4[3296]
50–59	38.6[5806]	26.1[3179]	15.9[432]	29.8[1473]	40.1[1695]	33.8[1883]	32.4[14468]
60–69	26.4[3968]	20.1[2456]	34.0[927]	21.7[1071]	29.2[1232]	23.4[1305]	24.5[10959]
70+	24.1[3633]	12.4[1515]	34.6[942]	40.1[1985]	21.7[915]	27.7[1544]	23.6[10534]
Mean age [sd]	60.3[±11.8]	50.0[±16.4]	63.1[±14.0]	62.0[±12.9]	60.3[±12.2]	60.0[±14.0]	57.8[±14.6]
Number of cases	15048	12198	2725	4946	4227	5571	44715
**Sex**							
Male	48.1[7181]	38.7[4717]	38.2[1041]	37.8[1867]	42.6[1798]	51.3[2851]	44.0[18414
Female	51.9[7749]	61.3[7481]	61.8[1684]	62.2[3067]	57.4[2427]	48.8[2712]	56.0[23436]
Number of cases	14930	12198	2725	4934	4225	5563	41850
**Place of residence**							
Urban	49.0[7376]	25.7[3132]	73.7[2007]	75.0[3709]	66.6[2810]	40.9[2280]	47.7[21314
Rural	51.0[7672]	74.3[9066]	26.4[718]	25.0[1237]	33.4[1411]	59.1[3289]	52.3[23393]
Number of cases	15048	12198	2725	4946	4221	5569	44707
**Marital status**							
Married	82.9[12366]	77.5[9456]	58.7[1533]	56.8[2797]	47.4[1965]	59.8[3308]	71.7[29892]
Not married	2.2[323]	5.7[689]	9.5[249]	4.0[196]	15.7[652]	2.9[159]	4.8[2019]
Others	39.2[1933]	16.8[2051]	31.8[831]	39.2[1933]	36.9[1531]	37.3[2062]	23.5[9799]
Number of cases	4926	12196	2613	4926	4148	5529	41710
**Education**							
No education	24.1[3629]	45.2[5080]	17.0[444]	1.0[44]	25.6[904]	51.0[2606]	30.3[12707]
Primary school or less	35.6[5358]	25.8[2894]	59.5[1555]	9.0[392]	46.5[1642]	22.9[1170]	31.1[13011[
Secondary school	35.1[5275]	23.1[2592]	14.4[377]	69.9[3042]	22.5[795]	22.4[1145]	31.6[13226]
Tertiary or higher	5.2[786]	5.9[664]	9.1[237]	20.1[876]	5.5[193]	3.6[185]	7.0[2941]
Number of cases	15048	11230	613	4354	3534	5106	41885
**Occupation**							
Public sector	41.8[5343]	9.7[714]	14.9[219]	86.2[3636]	16.4[562]	9.4[469]	31.9[10943]
Private sector	11.5[1466]	12.6[931]	29.8[437]	9.9[416]	55.4[1903]	4.2[207]	15.6[5360]
Self-employment	43.7[5592]	45.7[3374]	33.6[493]	2.5[106]	4.3[149]	79.5[3966]	40.0[13680]
Informal sector	3.0[385]	32.1[2372]	21.8[320]	1.5[61]	24.9[823]	7.0[347]	12.6[4308]
Number of cases	12786	7391	1469	4219	3437	4989	34291
**Household wealth**							
Q1 (Lowest)	19.1[2851]	20.7[563]	17.4[2114]	17.3[856]	19.4[814]	19.5[1081]	18.6[8279]
Q2	19.8[2960]	20.3[553]	19.1[2317]	19.2[946]	19.6[824]	19.8[1100]	19.6[8700]
Q3	20.1[2992]	18.8[511]	19.2[2323]	20.1[992]	19.0[798]	19.9[1104]	19.6[8720]
Q4	20.7[3088]	20.5[559]	21.1[2551]	20.7[1024]	20.7[871]	20.3[1126]	20.7[9219]
Q5 (Highest)	20.5[3058]	19.7[535]	23.2[2815]	22.7[1122]	21.3[896]	20.7[1148]	21.5[9574]
Number of cases	14949	2721	12120	4940	4203	5559	44492
C**urrent tobacco smoking**							
Never	17.4[837]	8.3[391]	50.2[492]	37.8[507]	24.0[339]	50.6[11.3]	22.0[3189]
Occasionally	7.7[368]	6.1[286]	18.3[179]	6.1[82]	11.8[167]	11.3[139]	8.4[1221]
Daily	74.9[3594]	85.6[4038]	31.5[309]	56.1[753]	64.1[905]	38.2[470]	69.5[10069]
Number of cases	4799	4715	980	1342	1411	1232	14479
**Ever alcohol intake**							
No	69.0[10062]	87.0[9767]	51.3[1341]	25.3[1092]	72.8[2929]	41.3[2102]	65.2[27293]
Yes	31.0[4513]	13.0[1457]	48.7[1272]	74.7[3217]	27.2[1096]	58.7[2987]	34.8[14542]
Number of cases	14575	11224	2613	4309	4025	5089	41835
**Fruits or vegetable intake**							
No or insufficient (<5 servings/day)	11.2[1581]	87.5[9816]	76.2[1969]	72.0[2688]	71.7[2874]	67.0[3385]	54.7[22313
Sufficient (>5 servings/day)	88.8[12585]	12.5[1406]	23.8[615]	28.1[1048]	28.3[1135]	33.0[1664]	45.3[18453]
	14160	11222	2584	3736	4009	5049	40766
**Chronic conditions**							
Hypertension (BP≥140/90mm/Hg)	28.6[4297]	17.0[2069]	33.4[873]	40.3[1993]	46.2[1951]	37.4[2081]	33.8[15116]
Diabetes	6.0[869]	4.9[550]	18.1[474]	8.3[357]	9.2[370]	3.5[177]	6.7[2797]
Stroke	3.1[456]	1.5[171]	4.3[112]	5.6[239]	3.6[144]	2.4[120]	3.0[1242]
Angina pectoris	6.7[1001]	5.5[667]	2.6[72]	25.8[1277]	4.9[205]	3.2[176]	7.6[3398]
Arthritis	17.9[2693]	16.7[2033]	13.5[367]	30.4[1505]	23.1[975]	19.3[1075]	19.3[8648]
Asthma	4.1[613]	7.5[910]	4.0[110]	6.2[308]	5.8[246]	3.2[178]	5.3[2365]
Chronic lung disease	8.7[1314]	11.0[1336]	12.4[337]	17.6[872]	6.1[256]	2.8[158]	9.6[4273]
Depression	2.0[299]	13.0[1570]	12.8[349]	6.4[316]	4.7[197]	7.7[428]	7.1[3159]
Low visual acuity	12.6[1896]	14.1[1715]	20.3[553]	15.8[783]	16.8[711]	13.9[776]	14.4[6434]
**Health insurance status**							
Without insurance	13.0[1935]	95.9[11697]	–	0.5[24]	81.4[3410]	63.5[3533]	49.3[20599]
With insurance	87.0[12987]	4.1[501]	–	99.5[4898]	18.6[780]	36.5[2029]	50.7[21195]
Number of cases	14922	12198		4922	4190	5562	41794

**Table 2:** Mean number of chronic conditions and prevalence of multimorbidity (%) by BMI categories and other characteristics in the six SAGE countries

Characteristics	China		India		Mexico		Russia		South Africa		Ghana		Pooled Countries	
	
	No of chronic conditions Mean [95%CI]	Prevalence of Multimorbidity [%,n]	Mean no of chronic conditions [95%CI]	Prevalence of Multimorbidity [%,n]	Mean no of chronic conditions [95%CI]	Prevalence of Multimorbidity [%,n]	Mean no of chronic conditions [95%CI]	Prevalence of Multimorbidity [%,n]	Mean no of chronic conditions [95%CI]	Prevalence of Multimorbidity [%,n]	Mean no of chronic conditions [95%CI]	Prevalence of Multimorbidity [%,n]	Mean no of chronic conditions [95%CI]	Prevalence of Multimorbidity [%,n]
Total	2.6[2.5,2.6]	21.5[3049]	2.6[2.5,2.6]	23.7[2583]	2.5[2.5,2.6]	27.4[717]	3.0[2.9,3.0]	49.6[2129]	2.6[2.5,2.7]	21.9[878]	2.4[2.3,2.4]	23.3[1156]	2.6[2.6,2.7]	22.6[9343]
**BMI status**														
Normal	2.5[2.5,2.6]	18.5[1575]	2.6[2.5,2.6]	21.8[1224]	2.5[2.4,2.7]	24.2[145]	2.8[2.7,3.0]	38.7[376]	2.5[2.4,2.6]	15.3[153]	2.4[2.3,2.5]	21.8[616]	2.6[2.5,2.6]	17.6[3440]
Underweight	2.4[2.3,2.6]	17.5[109]	2.5[2.5,2.6]	25.1[951]	2.3[1.7,3.0]	15.0[3]	2.3[1.7,2.8]	27.5[11]	2.8[2.4,3.2]	14.3[22]	2.3[2.2,2.4]	26.7[186]	2.5[2.4,2.6]	15.7[836]
Overweight	2.6[2.5,2.6]	25.7[950]	2.6[2.5,2.7]	26.2[307]	2.5[2.4,2.6]	25.2[245]	2.9[2.9,3.0]	45.4[718]	2.6[2.4,2.7]	19.5[211]	2.4[2.3,2.5]	22.9[216]	2.6[2.6,2.7]	26.7[2522]
Obese	2.6[2.5,2.7]	36.4[273]	2.6[2.5,2.8]	30.1[101]	2.5[2.4,2.6]	31.1[241]	3.1[3.0,3.2]	58.7[745]	2.7[2.6,2.8]	28.0[462]	2.4[2.3,2.6]	27.7[138]	2.8[2.8,2.9]	37.1[1960]
χ^2^ p value		<0.0001		<0.0001			0.007	<0.0001		<0.0001		0.004		<0.0001
**Age**														
18–29	–	1.0[2]	2.2[2.0,2.3]	1.9[31]	-	5.3[3]	-	2.2[2]	2.3[1.7,3.0]	3.2[3]	-	1.6[2]	2.1[2.0,2.3]	2.0[43]
30–39	2.2[1.8,2.5]	1.2[6]	2.2[2.1,2.3]	6.9[114]	2.2[1.9,2.5]	6.0[10]	2.2[1.8,2.7]	6.2[9]	-	1.7[2]	-	1.0[3]	2.2[2.1,2.3]	5.0[144]
40–49	2.2[2.1,2.4]	5.6[50]	2.4[2.3,2.6]	12.0[168]	2.2[2.0,2.3]	11.6[22]	2.3[2.2,2.5]	18.7[32]	2.9[2.4,3.3]	10.8[16]	2.1[2.0,2.3]	3.8[14]	2.4[2.3,2.5]	9.5[302]
50–59	2.4[2.3,2.4]	12.6[691]	2.5[2.5,2.6]	19.0[559]	2.2[2.0,2.3]	23.5[100]	2.7[2.6,2.8]	34.9[507]	2.6[2.5,2.7]	19.6[313]	2.3[2.2,2.4]	7.6[127]	2.5[2.5,2.5]	16.9[2297]
60–69	2.5[2.5,2.6]	26.4[994]	2.6[2.5,2.5]	26.8[597]	2.4[2.3,2.5]	29.1[26.5]	3.0[2.9,3.1]	56.3[595]	2.6[2.5,2.7]	26.4[310]	2.5[2.4,2.6]	14.1[169]	2.6[2.6,2.7]	28.3[2930]
70+	2.7[2.6,2.7]	39.4[1306]	2.7[2.6,2.8]	36.8[509]	2.5[2.4,2.6]	36.7[317]	3.2[3.1,3.3]	71.8[984]	2.5[2.4,2.6]	26.7[234]	2.4[2.3,2.5]	19.8[277]	2.8[2.8,2.8]	39.4[3627]
χ^2^ p value		<0.0001		<0.0001		<0.0001		<0.0001		<0.0001		<0.0001		<0.0001
**Sex**														
Male	2.4[2.4,2.4]	16.9[1099]	2.5[2.4,2.5]	13.8[585]	2.3[2.2,2.4]	15.2[136]	2.6[2.5,2.7]	37.3[522]	2.5[2.4,2.6]	14.5[240]	2.2[2.2,2.3]	5.4[142]	2.5[2.4,2.5]	15.7[2558]
Female	2.4[2.4,2.5]	19.8[1401]	2.4[2.3,2.4]	9.7[645]	2.4[2.4,2.5]	27.6[406]	2.8[2.8,2.9]	51.6[1273]	2.5[2.5,2.6]	23.7[530]	2.3[2.2,2.3]	9.7[224]	2.5[2.5,2.6]	19.6[4073]
χ^2^ p value		<0.0001		<0.0001		<0.0001		<0.0001		<0.0001		<0.0001		<0.0001
**Place of residence**														
Urban	2.5[2.4,2.5]	24.8[1638]	2.4[2.4,2.5]	14.7[405]	2.4[2.3,2.5]	24.8[427]	2.7[2.7,2.8]	48.1[1390]	2.6[2.5,2.6]	22.8[594]	2.3[2.2,2.4]	10.6[216]	2.6[2.5,2.6]	25.1[4670]
Rural	2.3[2.3,2.3]	12.4[875]	2.4[2.4,2.4]	10.1[825]	2.4[2.2,2.5]	17.8[115]	2.8[2.7,2.9]	41.5[405]	2.4[2.3,2.5]	13.7[176]	2.2[2.1,2.3]	5.1[150]	2.4[2.4,2.4]	12.1[2546]
χ^2^ p value		<0.0001		<0.0001		<0.0001		<0.0001		<0.0001		<0.0001		<0.0001
**Marital status**														
Married	2.4[2.4,2.4]	17.5[1978]	2.4[2.4,2.5]	11.1[943]	2.3[2.2,2.4]	19.4[272]	2.1[2.1,2.1]	15.6[1687]	2.6[2.5,2.7]	20.8[387]	2.3[2.3,2.4]	8.4[250]	2.3[2.3,2.3]	15.1[4608]
Not married	2.3[2.1,2.5]	8.5[24]	2.5[2.0,2.9]	1.8[11]	2.4[2.1,2.6]	17.8[41]	2.4[2.0,2.8]	24.5[12]	2.5[2.3,2.6]	17.1[105]	2.1[1.9,2.3]	6.4[9]	2.4[2.2,2.6]	5.0[47]
Others	2.5[2.4,2.7]	24.9[495]	2.4[0.4,2.5]	15.2[276]	2.5[2.4,2.6]	31.3[229]	2.8[2.7,2.9]	15.3[308]	2.6[2.6,2.7]	25.5[373]	2.4[2.4,2.5]	17.0[331]	2.6[2.5,2.6]	18.6[1079]
χ^2^ p value		<0.0001		<0.0001		<0.0001		<0.0001		<0.0001		<0.0001		<0.0001
**Education**														
No education	2.5[2.4,2.5]	21.2[641]	2.4[2.3,2.4]	10.7[527]	2.3[2.2,2.5]	22.3[90]	–	50.0[2]	2.5[2.4,2.6]	17.2[150]	2.4[2.3,2.5]	12.0[310]	2.4[2.4,2.5]	15.1[1260]
Primary school or less	2.4[2.4,2.4]	17.5[890]	2.5[2.4,2.5]	12.3[346]	2.5[2.4,2.5]	24.6[346]	2.9[2.7,3.1]	53.3[49]	2.6[2.5,2.7]	25.8[409]	2.4[2.3,2.5]	11.2[131]	2.4[2.4,2.5]	17.4[1631]
Secondary school	2.4[2.4,2.4]	17.2[840]	2.4[2.3,2.5]	10.8[272]	2.4[2.2,2.6]	16.2[56]	2.2[2.1,2.2]	14.9[1862]	2.7[2.5,2.9]	19.8[151]	2.4[2.3,2.5]	11.1[126]	2.3[2.2,2.3]	15.0[3030]
Tertiary or higher	2.4[2.3,2.6]	21.1[142]	2.4[2.3,2.6]	13.3[85]	2.3[2.1,2.5]	23.6[50]	2.7[2.5,2.9]	38.5[95]	2.7[2.4,3.0]	17.6[33]	2.6[2.2,2.9]	13.7[25]	2.5[2.4,2.6]	21.0[372]
χ^2^ p value		<0.0001		<0.0001		0.002		<0.0001		<0.0001		0.671		<0.0001
**Occupation**														
Public sector	2.7[2.6,2.7]	30.7[1561]	2.7[2.6,2.9]	21.1[150]	2.5[2.3,2.7]	25.1[55]	3.0[3.0,3.1]	52.2[1877]	2.8[2.6,2.9]	25.3[137]	2.5[2.3,2.7]	14.1[66]	2.8[2.8,2.9]	35.9[3846]
Private sector	2.6[2.5,2.7]	18.4[262]	2.6[2.5,2.7]	16.3[151]	2.4[2.3,2.6]	23.8[104]	2.7[2.6,2.9]	33.5[139]	2.5[2.5.2.6]	20.7[379]	2.5[2.2,2.7]	10.7[22]	2.6[2.5,2.6]	20.2[1057]
Self-employment	2.3[2.3,2.4]	15.5[844]	2.5[2.4,2.6]	15.9[535]	2.4[2.3,2.5]	25.6[126]	2.8[2.3,3.4]	22.9[24]	2.3[2.0,2.6]	18.6[27]	2.4[2.3,2.5]	12.0[474]	2.4[2.4,2.4]	15.0[2030]
Informal sector	2.5[2.3,2.7]	18.6[70]	2.5[2.5,2.6]	20.6[489]	2.4[2.2,2.6]	24.4[78]	2.9[2.5,3.3]	45.9[28]	2.5[2.4,2.7]	18.9[151]	2.1[2.0,2.1]	5.8[20]	2.5[2.5,2.6]	19.6[836]
χ^2^ p value		<0.0001		<0.0001		0.935		<0.0001		0.033		0.002		<0.0001
**Household wealth**														
Q1 (Lowest)	2.4[2.3,2.4]	18.9[493]	2.4[2.2,2.5]	8.2[159]	2.4[2.3,2.6]	18.0[88]	2.8[2.7,2.9]	48.0[320]	2.4[2.3,2.6]	10.6[77]	2.3[2.1,2.4]	4.0[38]	2.5[2.4,2.5]	15.9[1175]
Q2	2.4[2.4,2.5]	16.3[447]	2.4[2.3,2.5]	9.2[190]	2.5[2.3,2.6]	25.2[121]	2.8[2.7,2.8]	53.2[395]	2.4[2.3,2.6]	15.9[120]	2.2[2.1,2.3]	6.0[58]	2.5[2.5,2.6]	17.2[1331]
Q3	2.4[2.4,2.5]	19.9[541]	2.5[2.4,2.6]	10.1[211]	2.5[2.3,2.6]	25.4[112]	2.8[2.7,2.9]	49.7[377]	2.6[2.5,2.8]	22.9[176]	2.3[2.2,2.5]	5.5[54]	2.6[2.5,2.6]	19.0[1471]
Q4	2.4[2.3,2.5]	18.1[512]	2.4[2.3,2.5]	13.6[311]	2.4[2.3,2.5]	24.0[116]	2.7[2.6,2.8]	43.1[347]	2.5[2.4,2.7]	25.7[208]	2.2[2.1,2.3]	8.4[86]	2.5[2.4,2.5]	19.2[1580]
Q5 (Highest)	2.4[2.4,2.5]	18.9[509]	2.4[2.4,2.5]	14.5[357]	2.3[2.2,2.4]	22.0[104]	2.7[2.6,2.8]	39.9[354]	2.5[2.4,2.6]	22.9[186]	2.3[2.2,2.4]	12.8[130]	2.5[2.4,2.5]	19.7[1640]
χ^2^ p value		0.003		<0.0001		0.096		<0.0001		<0.0001		<0.0001		<0.0001
**Tobacco smoking**														
Never	0.5[2.4,2.5]	31.9[250]	2.6[2.4,2.8]	23.5[89]	2.3[2.2,2.4]	23.9[110]	2.9[2.8,3.1]	50.6[228]	2.5[2.3,2.7]	20.9[69]	2.3[2.1,2.5]	6.5[39]	2.6[2.5,2.7]	26.1[785]
Occasionally	2.4[2.2,2.5]	15.1[53]	2.6[2.3,2.8]	20.5[58]	2.6[2.3,2.9]	19.2[32]	2.5[2.2,2.9]	27.1[19]	2.4[2.1,2.8]	17.1[28]	2.1[1.9,2.3]	6.7[9]	2.5[2.4,2.6]	17.0[199]
Daily	2.3[2.3,2.4]	12.3[419]	2.5[2.4,2.5]	11.4[449]	2.4[2.2,2.6]	20.7[57]	2.5[2.4,2.7]	31.4[214]	2.4[2.3,2.6]	18.3[161]	2.2[1.8,2.5]	2.9[13]	2.4[2.4,2.5]	13.6[1313]
χ^2^ p value		<0.0001		<0.0001		0.282		<0.0001		0.731		<0.0001		<0.0001
**Ever alcohol intake**														
No	2.4[2.4,2.5]	19.6[1838]	2.4[2.4,2.4]	10.8[1022]	2.4[2.3,2.5]	23.5[285]	2.7[2.6,2.9]	49.4[478]	2.5[2.5,2.6]	21.3[601]	2.3[2.2,2.4]	7.0[143]	2.5[2.4,2.5]	16.9[4367]
Yes	2.4[2.3,2.4]	15.7[669]	2.5[2.4,2.6]	14.5[208]	2.4[2.3,2.5]	22.3[257]	2.8[2.7,2.8]	45.5[1317]	2.5[2.4,2.6]	15.8[168]	2.3[2.2,2.3]	7.7[223]	2.6[2.5,2.6]	20.7[2842]
χ^2^ p value		<0.0001		<0.0001		0.012		0.103		0.001		0.449		<0.0001
**Fruits or vegetable intake**														
No or insufficient (<5servings/day)	2.6[2.6,2.7]	25.5[394]	2.5[2.5,2.6]	17.6[1727]	2.5[2.5,2.6]	28.2[555]	3.0[2.9,3.1]	53.8[1444]	2.6[2.5,2.6]	21.1[603]	2.4[2.3,2.5]	12.0[406]	2.7[2.6,2.7]	23.1[5129]
Sufficient (>5servings/day)	2.5[2.5,2.6]	21.3[2608]	2.7[2.6,2.8]	17.8[250]	2.5[2.4,2.7]	26.0[160]	3.0[2.9,3.1]	46.8[486]	2.7[2.6,2.8]	24.0[269]	2.4[2.3,2.5]	11.0[183]	2.6[2.6,2.6]	21.1[3956]
χ^2^ p value		<0.0001		0.854		0.294		<0.0001		0.048		0.302		0.006
**Health insurance status**														
With insurance	2.5[2.4,2.6]	21.8[388]	2.4[2.4,2.4]	11.0[1148]	–	–	2.3[1.9,2.6]	35.0[7]	2.5[2.5,2.6]	19.5[614]	2.2[2.1,2.3]	4.9[156]	2.4[2.4,2.5]	12.4[2313]
Without insurance	2.4[2.4,2.4]	18.9[2111]	2.6[2.5,2.8]	18.7[82]	–	–	2.8[2.7,2.8]	46.5[1781]	2.5[2.3,2.6]	21.6[152]	2.3[2.2,2.4]	11.7[210]	2.5[2.5,2.6]	23.4[4336]
χ^2^ p value		<0.0001		<0.0001				0.299		0.054		<0.001		<0.0001

**Table 3:** Adjusted odds ratios showing the association between BMI status and non communicable disease multimorbidity by countries

Characteristics	China	India	Mexico	Russia	South Africa	Ghana	Pooled countries
	
	Adjusted OR[95%CI]	Adjusted OR[95%CI]	Adjusted OR[95%CI]	Adjusted OR[95%CI]	Adjusted OR[95%CI]	Adjusted OR[95%CI]	Adjusted OR[95%CI]
**BMI status**							
Normal	1[ref]	1[ref]	1[ref]	1[ref]	1[ref]	1[ref]	1[ref]
Underweight	0.57[0.32–1.01]	1.01[0.74–1.39]	0.08[0.01–0.90]	0.42[0.06–2.72]	2.31[0.76–7.05]	1.07[0.56–2.05]	1.23[0.93–1.63]
Overweight	1.81[0.92–3.53]	2.33[1.35–4.02]	0.39[0.16–0.97]	1.13[0.51–2.50]	0.69[0.26–1.83]	1.43[0.71–2.88]	1.47[1.05–2.07]
Obese	7.06[2.47–20.21]	1.99[0.79–5.03]	1.97[0.69–5.64]	9.90[3.90–25.17]	4.66[2.16–10.08]	5.61[1.21–26.02]	5.78[3.55–9.40]

Models are adjusted for age, sex, place of residence, marital status, education, occupation, household wealth, tobacco smoking, alcohol drinking, and fruits and vegetable intake. Multimorbidity is defined as the presence of two or more chronic conditions [angina pectoris, arthritis, asthma, low visual acuity, diabetes (excluding diabetes associated with a pregnancy), stroke, chronic lung disease, hypertension and depression]. ref- indicate reference category.

## References

[1] van den Akker M, Buntinx F, Knottnerus JA (1996). Comorbidity or multimorbidity: what’s in a name? A review of literature. Eur J Gen Pract.

[2] Fortin M, Bravo G, Hudon C, Vanasse A, Lapointe L (2005). Prevalence of Multimorbidity Among Adults Seen in Family Practice. The Annals of Family Medicine.

[3] Diederichs C, Berger K, Bartels D (2011). The measurement of multiple chronic diseases-a systematic review on existing multimorbidity indices. J Gerontol A Biol Sci Med Sci.

[4] Fortin M, Hudon C, Haggerty J, Akker M, Almirall J (2010). Prevalence estimates of multimorbidity: a comparative study of two sources. BMC Health Serv Res.

[5] Glynn LG, Valderas JM, Healy P, Burke E, Newell J, Gillespie P, Murphy AW (2011). The prevalence of multimorbidity in primary care and its effect on health care utilization and cost. Fam Pract.

[6] Vogeli C, Shields AE, Lee TA, Gibson TB, Marder WD, Weiss KB, Blumenthal D (2007). Multiple chronic conditions: prevalence, health consequences, and implications for quality, care management, and costs. J Gen Intern Med.

[7] Bayliss EF, Edwards AE, Steiner JF, Main DS (2008). Processes of care desired by elderly patients with multimorbidities. Fam Pract.

[8] Condelius A, Edberg AK, Jakobsson U, Hallberg IR (2008). Hospital admissions among people 65+ related to multimorbidity, municipal and outpatient care. Arch Gerontol Geriatr.

[9] Agborsangaya CB, Lau D, Lahtinen M, Cooke T, Johnson JA (2013). Health-related quality of life and healthcare utilization in multimorbidity: results of a cross-sectional survey. Qual Life Res.

[10] Pati S, Agrawal S, Swain S, Lee JT, Vellakkal S, Hussain MA (2014). Non-communicable disease multimorbidity and associated health care utilization and expenditures in India: cross-sectional study. BMC Health Serv Res.

[11] Britt HC, Harrison CM, Miller GC, Knox SA (2008). Prevalence and patterns of multimorbidity in Australia. Med J Aust.

[12] Stange KC (2012). In this issue: challenges of managing multimorbidity. Ann Fam Med.

[13] Dawes M (2010). Co-morbidity: we need a guideline for each patient not a guideline for each disease. Fam Pract.

[14] Hussain MA, Huxley RR, Mamun AA (2015). Multimorbidity prevalence and pattern in Indonesian adults: an exploratory study using national survey data. BMJ Open.

[15] Pati S, Swain S, Hussain MA, van den Akker M, Metsemakers J, Knottnerus JA, Salisbury (2015). Prevalence and outcomes of multimorbidity in South Asia: a systematic review. BMJ Open.

[16] Lee JT, Hamid F, Pati S, Atun R, Millett C (2015). Impact of Noncommunicable Disease Multimorbidity on Healthcare Utilisation and Out-Of-Pocket Expenditures in Middle-Income Countries: Cross Sectional Analysis. PLoS ONE.

[17] Agborsangaya CB, Lau D, Lahtinen M, Cooke T, Johnson JA (2012). Multimorbidity prevalence and patterns across socioeconomic determinants: a cross-sectional survey. BMC Public Health.

[18] Taylor AW, Price K, Gill TK, Adams R, Pilkington R, Carrangis N, Shi Z, Wilson D (2010). Multimorbidity - not just an older person’s issue: results from an Australian biomedical study. BMC Public Health.

[19] Sailer D (1998). Obesity: entrance port to multimorbidity]. Wiener medizinische Wochenschrift (1946).

[20] de Mutsert R, Sun Q, Willett WC, Hu FB, van Dam RM (2014). Overweight in early adulthood, adult weight change, and risk of type 2 diabetes, cardiovascular diseases, and certain cancers in men: a cohort study. Am J Epidemiol.

[21] de S Santos Machado V, Valadares AL, Costa-Paiva LH, Osis MJ, Sousa MH, Pinto-Neto AM (2013). Aging, obesity, and multimorbidity in women 50 years or older: a population-based study. Menopause.

[22] Agborsangaya CB, Majumdar SR, Sharma AM, Gregg EW, Padwal RS (2015). Multimorbidity in a prospective cohort: Prevalence and associations with weight loss and health status in severely obese patients. Obesity.

[23] Booth HP, Prevost AT, Gulliford MC (2014). Impact of body mass index on prevalence of multimorbidity in primary care: cohort study. Fam Pract.

[24] Must A, Spadano J, Coakley EH, Field AE, Colditz G, Dietz WH (1999). The disease burden associated with overweight and obesity. JAMA.

[25] World Health Organization (2009). Global Health Risks: Mortality and Burden of Disease Attributable to Selected Major Risks.

[26] World Health Organization (2010). Global Status Report on Noncommunicable Diseases 2010.

[27] Finucane MM, Stevens GA, Cowan MJ (2011). National, regional, and global trends in body-mass index since 1980: systematic analysis of health examination surveys and epidemiological studies with 960 country-years and 9.1 million participants. Lancet.

[28] Kowal P, Chatterji S, Naidoo N (2012). Data resource profile: the World Health Organization Study on global AGEing and adult health (SAGE). Int J Epidemiol.

[29] He W, Muenchrath MN, Kowal P (2012). Shades of gray: a cross-country study of health and well-being of the older populations in SAGE countries, 2007–2010.

[30] Naidoo N (2012). WHO Study on global AGEING and adult health (SAGE) Waves 0 and 1 - Sampling information for China, Ghana, India, Mexico, Russia, and South Africa. SAGE Working Paper No 5.

[31] Arokiasamy P, Uttamacharya U, Jain K, Biritwum RB, Yawson AE (2015). The impact of multimorbidity on adult physical and mental health in low- and middle-income countries: what does the study on global ageing and adult health (SAGE) reveal?. BMC Med.

[32] Whitworth JA (2003). World Health Organization/International Society of Hypertension statement on management of hypertension. J Hypertens.

[33] Peltzer K, Phaswana-Mafuya N (2013). Hypertension and associated factors in older adults in South Africa. Cardiovascular Journal of Africa.

[34] Chobanian AV, Bakris GL, Black HR, Cushman WC, Green LA (2003). Seventh Report of the Joint National Committee of Prevention, Detection, Evaluation, and Treatment of High Blood Pressure. JAMA.

[35] Arokiasamy P, Parasuraman S, Sekher TV, Lhungdim H (2013). Study on global AGEing and adult health (SAGE) Wave 1.

[36] Virgili G, Acosta R, Grover LL, Bentley SA, Giacomelli G (2013). Reading aids for adults with low vision. Cochrane Database Syst Rev.

[37] International Council of Ophthalmology (1984). Visual acuity measurement standard. Visual Functions Committee.

[38] Kessler RC, Ustun TB (2004). The World Mental Health (WMH) Survey Initiative Version of the World Health Organization (WHO) Composite International Diagnostic Interview (CIDI). Int J Methods Psychiatr Res.

[39] WHO (1993). The ICD-10 classification of mental and behavioural disorders: diagnostic criteria for research (DCR-10).

[40] Ayuso-Mateos JL, Nuevo R, Verdes E, Naidoo N, Chatterji S (2010). From depressive symptoms to depressive disorders: the relevance of thresholds. Br J Psychiatry.

[41] (2000). WHO Technical Report Series No 894. Obesity: Preventing and Managing the Global Epidemic. Report of a WHO Consultation.

[42] Moser KA, Agrawal S, Davey Smith G, Ebrahim S (2014). Socio-Demographic Inequalities in the Prevalence, Diagnosis and Management of Hypertension in India: Analysis of Nationally-Representative Survey Data. PLoS ONE.

[43] Ferguson BD, Tandon A, Gakidou E, Murray CJL, Murray CJL, Evans DB (2003). Estimating permanent income using indicator variables. Health Systems Performance Assessment: Debates, Methods and Empiricism.

[44] Hall JN, Moore S, Harper SB, Lynch JW (2009). Global variability in fruit and vegetable consumption. Am J Prev Med.

[45] Chatterji S, Kowal P, Mathers C, Naidoo N, Verdes E, Smith JP (2008). The health of aging populations in China and India. Health Aff (Millwood).

[46] WHO (1998). Guidelines for controlling and monitoring the tobacco epidemic.

[47] WHO (2012). WHO Study on Global Ageing and Adult Health (SAGE): core SAGE data and questionnaires. http://www.who.int/healthinfo/en/.

[48] Grimes D, Schultz K (2008). Making sense of odds and odds ratios. Obstetrics and Gynecology.

[49] Farrar DE, Glauber RR (1967). Multicollinearity in Regression Analysis: The Problem Revisited. The Review of Economics and Statistics.

[50] Oyebode O, Pape UJ, Laverty AA, Lee JT, Bhan N, Millett C (2015). Rural, Urban and Migrant Differences in Non-Communicable Disease Risk-Factors in Middle Income Countries: A Cross-Sectional Study of WHO-SAGE Data. PLoS ONE.

[51] Podestà F (2002). Recent Developments in Quantitative Comparative Methodology: The Case of Pooled Time Series Cross-section Analysis.

[52] Verma V, Gagliardi F, Ferretti C (2009). On Pooling of Data and Measures.

[53] Dong HJ, Unosson M, Wressle E, Marcusson J (2012). Health consequences associated with being overweight or obese: a Swedish population-based study of 85-year-olds. J Am Geriatr Soc.

[54] Guthrie B, Payne K, Alderson P (2012). Adapting clinical guidelines to take account of multimorbidity. BMJ.

[55] Wang F, Xu S, Shen X, Guo X, Shen R (2012). Epidemiology of multimorbidity. Lancet.

[56] Katzmarzyk PT, Reeder BA, Elliott S, Joffres MR, Pahwa P, Raine KD, Kirkland SA, Paradis G (2012). Body mass index and risk of cardiovascular disease, cancer and all-cause mortality. Can J Public Health.

[57] Ng M, Fleming T, Robinson M, Thomson B, Graetz N, Margono C (2014). Global, regional, and national prevalence of overweight and obesity in children and adults during 1980– 2013: a systematic analysis for the Global Burden of Disease Study 2013. Lancet.

[58] Diabetes Prevention Program Research Group (2002). Reduction in the incidence of type 2 diabetes with lifestyle intervention or metformin. N Engl J Med.

[59] The Look AHEAD Research Group (2010). Long-term effects of a lifestyle intervention on weight and cardiovascular risk factors in individuals with type 2 diabetes mellitus: four-year results of the Look AHEAD Trial. Arch Intern Med.

[60] Hosseinpoor AR, Bergen N, Mendis S, Harper S, Kunst A, Chatterji S (2012). Socioeconomic inequality in the prevalence of noncommunicable diseases in low- and middle-income countries: results from the World Health Survey. BMC Public Health.

[61] Basu S, King AC (2013). Disability and chronic disease among older adults in India: detecting vulnerable populations through the WHO SAGE Study. Am J Epidemiol.

[62] Levesque JF, Mukherjee S, Grimard D, Boivin A, Mishra S (2013). Measuring the prevalence of chronic diseases using population surveys by pooling selfreported symptoms, diagnosis and treatments: results from the World Health Survey of 2003 for South Asia. Int J Public Health.

[63] Allotey P, Davey T, Reidpath DD (2014). NCDs in low- and middle-income countries-assessing the capacity of health systems to respond to population needs. BMC Public Health.

[64] Goldman N, Lin IF, Weinstein M, Lin Y-H (2003). Evaluating the quality of self-reports of hypertension and diabetes. Journal of Clinical Epidemiology.

[65] Fortin M, Stewart M, Poitras M-E, Almirall J, Maddocks H (2012). A Systematic Review of Prevalence Studies on Multimorbidity: Toward a More Uniform Methodology. The Annals of Family Medicine.

[66] Vellakkal S, Subramanian SV, Millett C, Basu S, Stuckler D, Ebrahim S (2013). Socio-economic Inequalities in Non-Communicable Diseases Prevalence in India: Disparities between Self-Reported Diagnoses and Standardized Measures. PLoS ONE.

[67] The Italian Longitudinal Study on Aging Working G (1997). Prevalence of chronic diseases in older Italians: comparing self-reported and clinical diagnoses: the italian longitudinal study on aging working group. Int J Epidemiol.

[68] de Groot V, Beckerman H, Lankhorst GJ, Bouter LM (2003). How to measure comorbidity. A critical review of available methods. J Clin Epidemiol.

[69] Agrawal S, Ebrahim S (2011). Prevalence and risk factors for self-reported diabetes among adult men and women in India: Findings from a national cross-sectional survey. Public Health Nutri.

[70] Fortin M, Haggerty J, Almirall J, Bouhali T, Sasseville M, Lemieux M (2014). Life-style factors and multimorbidity: a cross sectional study. BMC Public Health.

[71] Ward BW, Schiller JS (2013). Prevalence of Multiple Chronic Conditions Among US Adults: Estimates From the National Health Interview Survey, 2010. Preventing Chronic Disease.

[72] Vellakkal S, Millett C, Basu S, Khan Z, Aitsi-Selmi A, Stuckler D (2015). Are estimates of socioeconomic inequalities in chronic disease artefactually narrowed by self-reported measures of prevalence in low-income and middle-income countries? Findings from the WHO-SAGE survey. J Epidemiol Community Health.

[73] Violan C, Foguet-Boreu Q, Flores-Mateo G, Salisbury C, Blom J, Freitag M (2014). Prevalence, determinants and patterns of multimorbidity in primary care: a systematic review of observational studies. PLoS One.

